# Medication Access in Prisons and Jails—Some Answers, More Questions

**DOI:** 10.1001/jamahealthforum.2023.0167

**Published:** 2023-04-07

**Authors:** Laura Hawks, Emily Wang

**Affiliations:** Division of General Internal Medicine, Medical College of Wisconsin, Milwaukee, Wisconsin; Yale School of Medicine, SEICHE Center for Health and Justice, New Haven, Connecticut

The landmark 1976 US Supreme Court case *Estelle v Gamble* conferred constitutional access to health care for people incarcerated in US prisons and jails. Specifically, the ruling outlawed “deliberate indifference” to the health needs of patients in carceral systems and defined this as delayed access to physicians for diagnosis and treatment, failure to administer treatment prescribed by a physician, and denial of professional medical judgment.^1^ However, the sparse external oversite of health care in carceral systems and a fractured collection of health data renders policymakers, advocates, and incarcerated patients and their family members virtually unable to monitor the quality of health care delivery within carceral walls. As a result, access to health care that meets appropriate standards of care remains far from guaranteed for incarcerated individuals in the US.

In this issue of *JAMA Health Forum*, Curran and colleagues^2^ take a novel approach to glean insight into the quality of care behind bars. They use data from the National Survey on Drug Use and Health and from IQVIA’s National Sales Perspective to estimate disease prevalence and access to pharmaceutical treatments, respectively, for 7 chronic conditions (diabetes, asthma, hypertension, hepatitis B and C, HIV, depression, and severe mental illness) in prisons and jails. They compared this access to the general population by measuring distribution of pharmaceutical treatments to carceral systems.

The authors estimated substantial underuse of medications in carceral systems compared with the general public given their respective prevalences of disease. These disparities ranged from 1.9- to 5.5-fold for each condition examined. The smallest difference in pharmaceutical distribution was among those with hepatitis C and the largest were among those with asthma. An analysis specific to type-2 diabetes treatment found a disparity compared with noncarceral settings in receipt of the most novel antidiabetes treatments (1.8% of all diabetes medications in prisons vs 12.3% outside of prisons), although indication could not be examined.

These national results are consistent with prior survey studies that have shown that medications to treat hepatitis C or opioid use disorder are largely underprescribed in prisons and jails.^3,4^ The study by Curran and colleagues^2^ suggests that such patterns exist at a national scale and across a number of common chronic medical and behavioral health conditions. However, the explanation for these findings is likely nuanced. Although the causes of undertreatment in prison settings in the absence of high-quality data may be difficult to discern, the reasons certainly expand beyond correctional leaders’ attitudes or individual institutional practices, as has been implicated as barriers to treatment for opioid use disorder.^4^

In fact, a host of policy-level barriers constrain carceral system pharmaceutical purchasing. A 2017 report issued by the Pew Foundation found that the processes by which correctional institutions acquire medications are complex and opaque.^5^ Only 11 state correctional systems were able to report the amount spent annually on pharmaceutical treatments. But almost all of these respondents reported that more than 15% of their system’s annual health care expenses were devoted to pharmaceutical purchases—which is higher than the national average of 10% of health care spending. This difference may be due to facing higher drug prices. In fact, state prisons and local jails buy many of the same drugs as Medicaid agencies because individual entities do not have substantial negotiating power. Further, because federal law excludes Medicaid from covering care in prisons and jails, they are not eligible for the federal Medicaid Drug Rebate Program, which effectively lowers drug manufacturer prices.

However, other pathways can support more affordable drug purchasing. Again, in the same Pew Foundation report, the Texas prison system reported the lowest proportional health care budget spent on pharmaceuticals due to partnerships, which allowed it to take advantage of the federal 340B Drug Discount Program.^5^ Importantly, Curran and colleagues^2^ found that the lowest disparity in drug distribution was in hepatitis C medications, which are among the most expensive single medications for treating a chronic health condition. This surprising finding may be attributable to states establishing collective purchasing agreements to obtain these medications at substantially lower costs.^6^

Perhaps most notably, this study^2^ further illuminates the paucity of data on the health and health care available to those incarcerated in US prisons and jails. Although the authors make important attempts to estimate prescription drug availability behind bars, no uniform system currently exists to measure the quality of care and the availability of medications in the health care systems of prisons and jails. This lack of data reveals a pernicious indifference to the health of individuals with criminal legal involvement. Most national population-based health studies exclude participants who are incarcerated.^7,8^ A decade and a half passed between data collections for the National Survey of Prison Inmates,^9^ and the most recent survey in 2016 eliminated most questions on the quality of health care. Without such data, carceral systems are rarely held accountable to uphold the health care mandate from the Supreme Court’s *Estelle v Gamble* ruling.

The findings of Curran and colleagues^2^ are important especially given that undertreatment of medical and behavioral health conditions in carceral settings extends beyond individual-level health outcomes. For example, public health experts consider prisons and jails a crucial target for achieving the United Nations’goal of hepatitis C virus eradication by 2030 given the high prevalence in such settings.^10^ The study suggests an important missed opportunity to achieve this goal. In addition, undertreatment of any chronic condition behind bars, given the disproportionate incarceration of racially minoritized populations, has broader implications for health disparities at large. Efforts to achieve health equity in the US will fail if these factors are not reversed.

To mitigate disparities in the use of prescription medications in the carceral setting and improve quality of care, correctional systems must be able to purchase drugs at lower costs similar to other systems that provide care to low-income people. Leveraging the purchasing power of 340B plans or Medicaid, as seen with hepatitis C medications, may improve pharmaceutical access in correctional systems. As evidenced by the current study,^2^ measures are needed to ensure the constitutional rights of incarcerated people in the US to appropriate health care.

## References

[1] Elger BS. Medical ethics in correctional healthcare: an international comparison of guidelines. J Clin Ethics. 2008;19(3):234–248. doi:10.1086/JCE200819305 19004433

[2] Curran J, Saloner B, Winkelman TNA, Alexander GC. Estimated use of prescription medications among individuals incarcerated in jails and state prisons in the US. JAMA Health Forum. 2023;4(4):e230482. doi:10.1001/jamahealthforum.2023.0482 37058293 PMC10105311

[3] Beckman AL, Bilinski A, Boyko R, New hepatitis C drugs are very costly and unavailable to many state prisoners. Health Aff (Millwood). 2016;35(10):1893–1901. doi:10.1377/hlthaff.2016.0296 27702964

[4] Nunn A, Zaller N, Dickman S, Trimbur C, Nijhawan A, Rich JD. Methadone and buprenorphine prescribing and referral practices in US prison systems: results from a nationwide survey. Drug Alcohol Depend. 2009;105(1-2):83–88. doi:10.1016/j.drugalcdep.2009.06.015 19625142 PMC2743749

[5] Huh K, Boucher A, McGaffey F, McKillop M, Schiff M. Pharmaceuticals in State Prisons: How departments of corrections purchase, use, and monitor prescription drugs. Pew Chartiable Trusts. 2017. Accessed February 13, 2023. www.pewtrusts.org/~/media/assets/2017/12/pharmaceuticals-in-state-prisons.pdf

[6] Auty SG, Griffith KN, Shafer PR, Gee RE, Conti RM. Improving access to high-value, high-cost medicines: the use of subscription models to treat hepatitis C using direct-acting antivirals in the United States. J Health Polit Policy Law. 2022;47(6):691–708. doi:10.1215/03616878-10041121 35867531 PMC9789167

[7] Ahalt C, Binswanger IA, Steinman M, Tulsky J, Williams BA. Confined to ignorance: the absence of prisoner information from nationally representative health data sets. J Gen Intern Med. 2012;27(2):160–166. doi:10.1007/s11606-011-1858-7 21922160 PMC3270223

[8] Wang EA, Aminawung JA, Wildeman C, Ross JS, Krumholz HM. High incarceration rates among black men enrolled in clinical studies may compromise ability to identify disparities. Health Aff (Millwood). 2014;33(5):848–855. doi:10.1377/hlthaff.2013.1325 24799583 PMC4065793

[9] Bureau of Justice Statistics. 2016 Survey of Prison Inmates (SPI) Questionnaire. Accessed February 13, 2023. https://bjs.ojp.gov/sites/g/files/xyckuh236/files/media/survey/spi16q_2.pdf

[10] Akiyama MJ, Kronfli N, Cabezas J, ; International Network on Health and Hepatitis in Substance Users–Prisons Network. Hepatitis C elimination among people incarcerated in prisons: challenges and recommendations for action within a health systems framework. Lancet Gastroenterol Hepatol. 2021;6(5):391–400. doi:10.1016/S2468-1253(20)30365-4 33857445 PMC8118192

